# Supplementary material for: 8-port MIMO antenna at 27 GHz for n261 band and exploring for body centric communication
Source: PLoS One. 2024 Jun 20;19(6):e0305524. doi: 10.1371/journal.pone.0305524 (PMC11189201; doi:10.1371/journal.pone.0305524)
Supplement: Attachment — Submitted filename: Plos One review response2.docx [file pone.0305524.s003.docx]

PONE-D-23-35854

8-port MIMO Antenna at 27 GHz for n261 band and exploring for Body Centric Communication

**Dear Editor and Reviewers,**

First of all, we would like to take this opportunity to express our sincere thanks to the editor and reviewers for their valuable time to make suggestions to improve the quality of this paper. We have revised the manuscript in accordance to the editor’s comments, and the changes in the revised manuscript are highlighted by yellow.

**Editor Comments**

**Comment 1: New comment: Incorporate SMA connector and compare with measured data?**

**Response 1:** Authors have incorporated the SMA connector and simulation and Figures 20 and Figure 21 are updated with the new results.

After adding the SMA connector, measured and simulated results are approximately overlapped with negligible discrepancy.

Antenna with incorporated Connectors while simulation

(a)

(b)

Figure 20 (a) with connector (b) without connector

(b)

Figure 21 (a) with connector (b) without connector

**Comment 2: There are so many unnecessary references that need to be eliminated. Editor is highlighting that please eliminate even if the reviewers recommended. It is completely irrelevant.**

**Response 2:** Thanks for the comments. We have removed the references irrelevant to the article's topic.
